# Disinfection of raw wastewater and activated sludge effluent using Fenton like reagent

**DOI:** 10.1186/s40201-014-0149-8

**Published:** 2014-12-14

**Authors:** Hassan Aslani, Ramin Nabizadeh, Mahmood Alimohammadi, Alireza Mesdaghinia, Kazem Nadafi, Reza Nemati, Maryam Ghani

**Affiliations:** Department of Environmental Health Engineering, School of Public Health, Tehran University of Medical Sciences, Tehran, Iran; Center for Air Pollution Research, Institute for Environmental Research, Tehran University of Medical Sciences, Tehran, Iran; Department of Environmental Health Engineering, School of Public Health, Shahid Beheshti University of Medical Science, Tehran, Iran

**Keywords:** Disinfection, Raw wastewater, Effluent, Modified Fenton, Cu^++^ ions

## Abstract

**Background and objectives:**

Water shortage problems have led to find either new water resources or improve wastewater treatment technologies in order to reuse. Due to less performance of previous units in microbial removal, disinfection has become a necessary step in wastewater treatment plants. In the present study performance of hydrogen peroxide (HP) and modified Fenton’s reagent (HP + Cu^++^) was considered for the disinfection of raw wastewater (RW) and activated sludge effluent (ASE).

**Materials and methods:**

Plastic containers of 10-liter volume each were used for RW and ASE sampling. Microbiological analyses of the RW and ASE were performed in triplicate; before and after the disinfection process. Fecal coliforms were analyzed by the direct (without enrichment) multiple fermentation tube procedure.

**Results:**

The results showed that using HP alone did not have any significant disinfection capability. In RW and ASE, the highest dose used in this study could reduce fecal coliforms (FC)_s_ by only 1.54 and 1.16 log-inactivation, respectively. However, Maximum removal efficiency of modified Fenton in RW and ASE was 4.63 and 3.41 log-inactivation, respectively. The results suggested that Cu^++^ ions used in combination with H_2_O_2_ produced very rigorous synergistic effect, and HP disinfection capacity increased significantly.

**Conclusion:**

Hydrogen peroxide, when applied alone, was not successful in disinfecting of either RW or ASE, and neither the WHO guideline nor the Iranian standard could be met. However, modified Fenton showed very significant disinfection potential and could reduce FC_s_ under World Health Organization (WHO) guideline and Iranian national standard for agricultural irrigation.

## Introduction

Nowadays, most countries have water shortage problems and it is the main reason that scientists are looking for new water resources and also try to develop new technologies to reuse treated and, in some cases, untreated wastewater as a water resource. Using untreated wastewater is not common in developed countries; but, in some poor and developing countries because of the lack of provisions by the authorities, people use untreated wastewater for agricultural irrigation or some other uses such a washing clothes; such usage would be considered as an important source for exposing people to some water-related diseases. According to WHO, wastewaters containing less than 1000 CFU (colony forming unit)/100 ml of fecal coliforms (FC) and no more than 1 helminthes Egg/l can be safely used for irrigation purposes [1,2].

Different units (i.e. physco-chemical and biological) of wastewater treatment plants attempt to remove pathogenic microorganisms to some extent; however, in the case of microorganism’s removal, there are no specific physco-chemical or biological processes which could provide qualified effluent of category A, defined by World Health Organization. Therefore, final disinfection is an obligatory step in wastewater treatment, especially when final effluents are to be re-used [1-3]. Different kinds of disinfectants have been used for many years (e.g. chlorine, chlorine dioxide, ozone, uv irradiation, etc.). Although application of the mentioned disinfectants have been proved to be effective, there are still some practical limitations. It has been proven that using chlorine as a disinfectant can produce harmful disinfection by-products (DBPs); in addition, the problem of storage and safe handling has led recent studies to look for alternative disinfectants [1,3-6]. In the last few decades, hydrogen peroxide (HP) has been introduced as an environmentally friendly disinfectant for wastewater disinfection. It has disinfection capability and does not leave any unfavorable environmental effects [5,7]. HP has weak disinfection capability by itself; however, recent research has shown that metallic cations such as silver (Ag^+^) and iron (Fe^++^) have a synergistic effect on HP disinfection potential [1-3,5,8-14]. The main disinfection mechanism in the removal of microorganisms by HP is free OH^•^ radical production; so, it is conceivable that when HP is combined with other metallic ions, more radicals are produced. Combining HP with ferrous iron such as Fe^++^ results in an oxidation system which is called Fenton’s reaction [1,3,10,15]; also, when HP is combined with other ions such as copper (cu^++^), the system is known as modified Fenton’s or Fenton-like reaction [16].

The main objective of the present study was to investigate the feasibility of modified Fenton’s reagent for the disinfection of RW and ASE. The results were compared with the effectiveness of HP alone, copper ions alone, and also the previous results that investigated Fenton’s reagent performance in the disinfection of similar test samples under similar conditions. It should be noted that studying raw wastewater disinfection was essential in the present study, since many treatment plants, especially in developing countries, try to bypass the untreated influent in some occasions such as hydraulic overflow during rainy periods and failure of treatment processes, which cause microbial contamination in receiving water bodies.

## Experiments

The materials used in the experimental set up included copper chloride dihydrated, hydrogenperoxide 30% solution, sodium chloride, sodium thiosulfate pentahydrate, and A1-Medium. All the chemicals used in this study were purchased from Merck Company. The necessary stock solutions were made from the above materials and doubled distilled water. The glassware was washed daily and autoclaved at 121°C for 15 min before use.

### Sampling and preparing the samples

Plastic 10-lit volume containers were used for RW and ASE sampling. The samples were taken from a municipal wastewater treatment plant in an activated sludge process located in the north of Tehran, Iran, and were immediately transported to the laboratory within an hour, where they were analyzed on a daily basis. The samples were taken from wastewater entering to the plant, and before disinfection unit for RW and ASE, respectively.

### Procedure

The study was conducted in three phases as mentioned in Table 1 at the ambient temperature ranging from 21 to 26°C. One hundred and twenty nine experiments were totally done in the laboratory. The selected doses of the reacting substances are also shown in Table 1. Experimental phase relating to different concentrations of HP and Cu^++^ included 9 and 7 tests, respectively. Doses of the reacting substances were increased gradually to determine the trend in disinfection practice. The research was carried out in the model laboratory reactors with the volume of 1 liter. A series of reactors was supplied with the RW and ASE at the same time and the chemical reagents at different concentrations were added. In the third phase, modified Fenton’s reagent was prepared at a different weigh ratios of cu^++^ to HP and added to the reactors. Samples were mixed thoroughly for 1 min and, after 30 min contact time disinfectant activity was stopped by adding a mole-to-mole ratio of sodium thiosulfate as neutralizer. Comparison tests were carried out using only HP and Cu^++^ ions. The difference between the initial and final (after exposure to disinfectant) FC number was used to evaluate disinfection efficacy.

**Table 1:** **Experimental phases and applied doses of reacting substances**

**No.**	**Phase1**	**Phase2**	**Phase3**	
	**H**_**2**_ **O**_**2**_ **(mg l** ^**−1**^ **)**	**Cu** ^**++**^ **(mg l** ^**−1**^ **)**	**Fenton Reagent**	
			**Cu** ^**++**^ **(mg l** ^**−1**^ **)**	**H**_**2**_ **O**_**2**_ **(mg l** ^**−1**^ **)**
1	100	0.01	0.5,1,2,5	100
2	200	0.1	0.5,1,2,5	200
3	300	0.5	0.5,1,2,5	300
4	400	1	0.5,1,2,5	400
5	500	2	0.5,1,2,5	500
6	600	2.5	0.5,1,2,5	600
7	700	5	0.5,1,2,5	700
8	800		0.5,1,2,5	800
9	900		0.5,1,2,5	900

CT factor is used for evaluation and comparison of different phases. In this formula, C is the initial concentration of applied material and T is contact time in minute. Therefore, according to other studies, CT is calculated by multiplying the initial used concentration (C) by contact time (T).

### Microbiological analysis

Microbiological analyses of the RW and ASE were performed in triplicate before and after the disinfection process according to the doses shown in Table 1. Fecal coliforms were analyzed by direct (without enrichment) multiple fermentation tube procedure (Standard Methods, 9221E-2). The samples were inoculated and incubated on A1 medium and their ability to produce gas and turbidity was determined. For the enumeration of FC bacteria, the samples were incubated at 37°C for 3 h and the tubes were then transferred to a 44.5°C water bath for 19–21 h.

## Results and discussion

The physico-chemical and microbiological characteristics of RW and ASE during the period of this research are shown in Table 2. It can be clearly seen that treating municipal wastewater with activated sludge process could reduce COD, BOD, TSS, and FCs by 88.8, 89.9, 91, and 94%, respectively. These figures show that wastewater treatment plants had significant efficiency in terms of removing different pollutants; however, especially in the case of FC reduction and health-related risks, it was not enough. Therefore, it is obvious that, by solely using biological treatment processes, the needed standards for re-use purposes cannot be met.

**Table 2:** **Physico-chemical and microbiological characteristics of RW and ASE**

**Parameter**	**Unit**	**RW**	**ASE**
COD	mg/L	580 ± 98	65 ± 32
BOD	mg/L	347 ± 26	35 ± 16
TSS	mg/L	769 ± 150	68 ± 20
pH	unit less	7.61 ± .34	7.3 ± 14
FCs	MPN/100 mL	9.2E + 06	5.5E + 05

**Figure 1:**
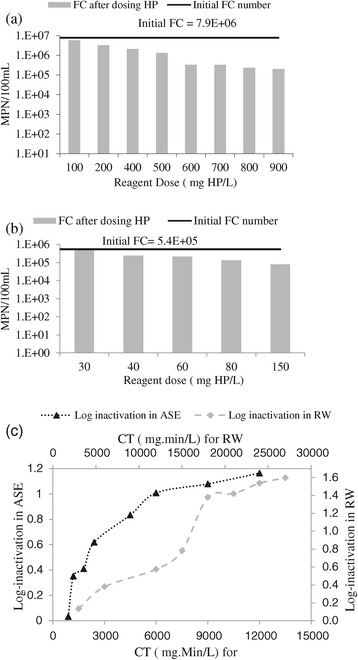
**Reduction of fecal coliforms in phase 1 of the experiments; in RW (a), in ASE (b), and log inactivation vs the CT factor using HP (c).**

Disinfection performance of the phase 1 of the experiments (H_2_O_2_ alone) is shown in Figure 1. Results obtained in this phase demonstrated that the used HP in the range of 100 and 900 mg/L did not have significant efficiency. Figure 1(a) and (b) demonstrate that the number of FCs decreased regularly by increasing HP dose. In the case of RW, using HP between 100 and 900 mg/L led to 0.13 and 1.6 log-inactivation, respectively (Figure 1c). Maximum reduction of FCs was related to the highest dose of HP; i.e. 900 mg/L. In the case of ASE, maximum FC removal (1.16 log-inactivation) occurred in CT value of 12000. The results showed that HP, at the concentrations of less than 30 mg/L, there was no disinfecting effect on ASE. According to Figure 1(c), it is clear that HP did not have the same behavior in RW and ASE. In CT value of 6000, 1.01 log-inactivation was achieved in ASE disinfection, while, for RW disinfection, the removal efficiency based on log-inactivation was 0.38 in the same CT. There was more oxidizing agent in RW than ASE, which would probably be the main reason for this behavior. Also, in ASE, there was a sharp increase in FC removal between the two first applied doses (CT values 900 to 1200) of HP and, afterward, the trend of removal was steady; In contrast, in the RW, there were three different parts: at first, the removal trend was slow, then the sharp slope happened in the middle (CT values 15000 to 18000) of the process when other oxidizing agents were oxidized by HP, and in the 3rd part, although the removal was upward, intensity fell back again. It is clear that in both cases (RW and ASE) after a sharp increase of FC removal, there was a steady increase until the end of the process. These figures clearly represent that HP alone did not have a significant disinfection effect, which was in good agreement with findings of other studies [3,10,13,17].

**Figure 2:**
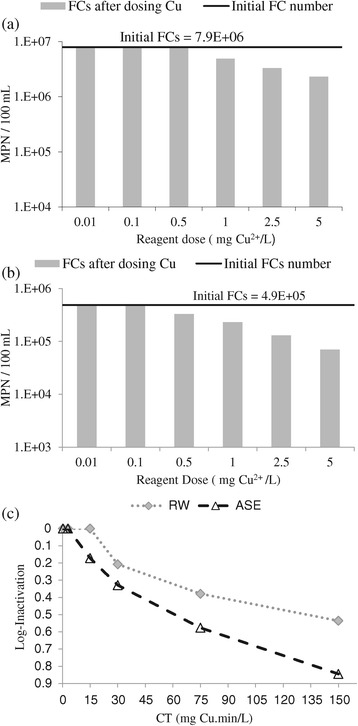
**Reduction of fecal coliforms in phase 2 of the experiments; in RW (a), in ASE (b), and log inactivation vs the CT factor using Cu** ^**++**^ **ions (c).**

Disinfection performance of phase 2 of the experiments (Cu^++^ alone) is shown in Figure 2. According to Figure 2(a), the initial number of FC_s_ in RW was 7.9E + 06 MPN/100 mL and applied doses of Cu^++^ did not show any significant disinfecting performance. At the first three stages of the process, removal efficiency was zero; at the final three stages, the number of FCs decreased slowly. The highest applied dose of Cu, i.e. 5 mg/L, reduced the number of FCs to just 2.30E + 06 MPN/100 mL. In the case of ASE (Figure 2(b)), the initial number of FC_s_ was 4.9E + 05 MPN/100 mL and the removal trend of FC_s_ was sharper than RW. The highest dose of Cu^++^ could reduce the survived number of FC_s_ to 7.00E + 04 MPN/100 mL. In addition, Cu^++^ at the concentrations of less than 0.5 mg/L did not show any notable disinfection efficiency. According to Figure 2(c), the trend of FC removal in ASE was somehow more severe (1.6 times higher) than that in RW and maximum removal efficiency in RW and ASE was 0.53 and 0.85 log-inactivation, respectively. It can be interfered from Figure 2(c) that the initial number of FC_s_ and amount of oxidizing agents such as dissolved organic matter are the important factors that influence a disinfectant’s performance.

**Figure 3:**
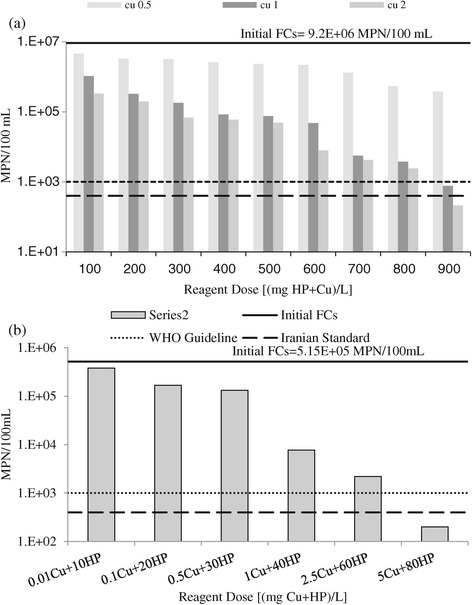
**Fecal coliform’s removal in phase 3 of the experiments; in RW (a), and in ASE (b).**

Disinfection performance of phase 3 of the experiments (HP + Cu^++^) is shown in Figure 3. In the case of RW, in this phase of the experiments, there were three different steps: in the 1st step, the combination of different doses of HP in the range of 100 to 900 mg/L and 0.5 mg/L Cu^++^ was used; the 2nd stage was related to the combination of 1 mg/L Cu^++^ and different doses of HP; and the last step was related to combining 2 mg/L Cu^++^ and HP (Figure 3(a)). According to this figure, the initial concentration of FC_s_ was 9.20E + 06 MPN/100 mL and the combination of 0.5 mg/L Cu^++^ and 900 mg/L HP could reduce the survived number of FC_s_ to just 3.8E + 05 MPN/100 mL. Also, removal trend of FC_s_ in the last two doses of HP was sharper than the previous one. Therefore, it is obvious that the combination of 0.5 mg/L Cu^++^ and different doses of HP did not show significant disinfecting performance, which could not have any synergistic effect on HP performance. It is clear that the WHO guideline, i.e. 1000 MPN/100 mL, or Iranian standard, i.e. 400MPN/100 mL, could not be met. Adding 1 mg/L Cu^++^ to HP showed significant disinfecting performance; although it could not reduce the survived FC_s_ to below the Iranian standard required for agricultural irrigation, the WHO guideline could be met. The final step in this phase showed great performance. It can be observed that, in the highest applied dose of reagents, i.e. 900 mg/L HP and 2 mg/L Cu^++^, both the WHO guideline and Iranian standards could be met.

Figure 3(b) shows the result of phase 3 of the experiments on ASE. The initial number of FC_s_ in the ASE which was used for disinfection analysis was 5.1E + 05 MPN/100 mL. Different doses of HP and Cu^++^ were combined and evaluated (see Figure 3(b)), representing that, by combining 80 mg HP/L and 5 mg Cu^++^/L, it was possible to reduce the number of FC_s_ to below the WHO guideline and Iranian Standard.

**Figure 4:**
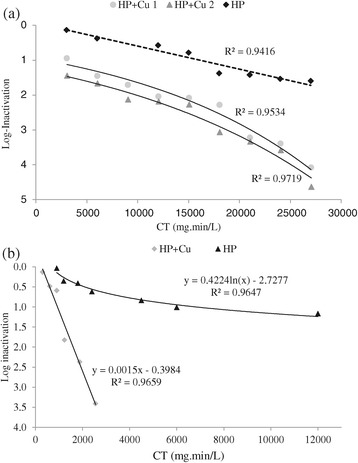
**Comparing disinfection performances of Hp and modified fenton’s reagent in (a) RW AND (b) ASE.**

**Figure 5:**
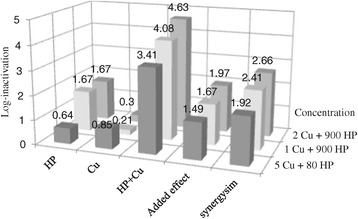
**Synergistic effect of Cu** ^**++**^ **on HP performance for disinfection of ASE and RW.**

Figure 4 shows log-inactivation versus CT value using modified Fenton’s reagent and HP in order to disinfect RW and ASE. It is clear that the combination of HP and Cu^++^ had more disinfection potential than HP alone. Comparison of HP and combination of HP and Cu^++^ are shown in the figure; as illustrated, HP alone showed a mild disinfection potential; however, in combination with Cu^++^, efficiency increased rapidly. It can be concluded that the combination of different doses of HP and 2 mg Cu^++^/L had the best performance for RW; for example, in CT value of approximately 27000, the combination of 1 and 2 mg/L Cu^++^ and 900 mg/L HP showed 4.08 and 4.63 log-inactivation, respectively, whereas using HP alone only demonstrated 1.6 log-inactivation in the same CT value. As noted above, using Cu^++^ at the concentrations of 0.5 mg/l or less did not show any synergistic effect; however, using concentrations equal to 1 mg/l or more demonstrated a significant synergistic effect. For example, applying 900 mg/L HP in combination with 1 and 2 mg/L Cu^++^ could increase efficiency of HP by 2.41 and 2.66, respectively (see Figure 5). Results of this part of the experiments were in agreement with ORTA De Velasquez et al.;s work; they used a combination of HP and Cu^++^ in order to disinfect advanced primary effluent treatment and, in CT value 30000, they could reduce FC_s_ by more than 6 log-inactivation. Moreover, it can be interfered from Figure 4(a) that using HP as the only disinfectant of RW (in the CT value of 24000) only showed 1.54 log-inactivation, while HP and Cu^++^ in combination could reach 3.58 log-inactivation in the CT value of 24060. Therefore, it could be good proof for copper synergistic effect. According to Pedahzure et al. [17], it seems that the synergistic effect of Cu^++^ was due to the stress conditions exerted by the combination of the two disinfectants rather than the formation of any highly active species known to develop during the HP reaction or multiple transition valences of the metal ions Fe^2+^, Fe^3+^, and Cu^1+^, Cu^2+^. In comparison to Fenton’s reagent (HP + Fe^++^) which was used in the previous study by the present authors, it is clear that modified Fenton’s reagent (HP + Cu^++^) was a more potent disinfectant than the former. In RW Fenton’s reagent in the CT value of 18750, i.e. 500 mg/L HP plus 125 mg/L Fe^++^ led to 1.54 log-inactivation [1], whereas modified Fenton in CT values 18030 and 18060 (i.e. combination of 600 mg/L HP with 1 and 2 mg/L Cu^++^) showed 2.28 and 3.07 log-inactivation, respectively.

According to Figure 4(b), it is obvious that, when HP was applied as the only disinfectant, the number of FC_s_ decreased by a logarithmic order, whereas applying a combination of HP and Cu^++^ led to the linear removal of FC_s_. In addition, the results showed that synergistic effect increased by increasing the concentration of HP and Cu^++^; in other words, synergistic effect increased by lowering HP to Cu^++^ ratio. Using modified Fenton’s reagent in the CT value of 2550, i.e. 80 mg/L HP plus 5 mg/L Cu^++^, 3.41 log-inactivation could be achieved, while using HP alone in the CT value of 2400 (related to 80 mg/L HP) only demonstrated 0.64 log-inactivation (see Figure 1(b)). Then, again, it is clear that Cu^++^ had a very high synergistic effect on HP performance; when combined, the overall efficiency was 3.41 log-inactivation; 1.92 of which was related to the synergistic effect of Cu^++^ (see Figure 5). In the case of ASE, again it was determined that disinfection performance of modified Fenton was more severe than that of Fenton’s reagent; e.g. according to the previous study [1], disinfection of ASE by Fenton’s reagent had 2.03 log-inactivation in the CT value of 15000; i.e. combination of 400 mg/L HP and 100 mg/L Fe^++^, whereas modified Fenton showed 2.37 log-inactivation in the CT value of just 1875 which was related to the combination of 60 mg/L HP and 2.5 mg/L Cu^++^.

Synergistic effect of Cu^++^ ions on HP disinfection performance is shown in Figure 5. According to this figure, it is obvious that the combination of 1 and 2 mg/L Cu^++^ ions with the fixed dose of HP, i.e. 900 mg/L, showed different synergistic effects of 2.41 and 2.66, respectively; then, it is clear that the synergistic effect was increased by increasing Cu^++^ concentration. On the other hand, it can be observed that the combination of 80 mg/L HP (minimum dose of HP used) and 5 mg/L Cu^++^ (maximum used dose) had a good synergistic effect, which was enough for FC_s_ removal in ASE. Therefore, as Fenton’s reagent needed the HP doses of 2 to 10 times of Fe^++^ for the best performance, finding a fair ratio of HP and Cu^++^ combination seemed necessary. According to the WHO guideline for Cu^++^ in drinking water, healthy precautions must be taken into account; concentrations of above 5 mg/L imparts color and an undesirable bitter taste to water. Also,, WHO health-based guideline for Cu^++^ is 2 mg/L [18]. The following studies using HP in combination with different kinds of metallic ions, such as iron, copper, and silver showed that, the same as chlorine and chloramines, it can provide a long-lasting residual biocidal action in the final effluent. Also, according to the previous studies [2,4,13,19,20], this new commercially stabilized available combination does not produce any disinfecting by-products and is effective in controlling biofilms in water distributing pipes.

## Conclusion

In some of the developing countries and also in countries with water shortage problems, using wastewater as a water resource for irrigation can compensate for most of the difficulties. However, its use poses adverse health hazards due to the high number of pathogenic organisms in wastewater. Although the secondary treatment of wastewater can reduce and remove most of the unwanted elements and produce a good-quality effluent in terms of nutritional constituents, to ensure microbiological quality, final disinfection is required. There are many conventional disinfectants such as chlorine, ozone, etc.; but, due to their numerous disadvantages, finding a ’non-conventional’ disinfectant with a long-lasting effect was considered in this research. In this regard, very satisfactory results were obtained by the follow-up treatment using HP in combination with copper.

Hydrogen peroxide, when applied alone, was not efficient in disinfecting either RW or ASE; morever, neither the WHO guideline nor Iranian standard could be met.

However, addition of Cu^++^ turned out to be very effective, as in RW, the CT value of 27060 reduced FC numbers by 4.63 log-inactivation, whereas using HP alone in the same CT value just showed 1.6 log-inactivation. In the case of ASE, the results were more reasonable and, in the CT value of 2550, 3.41 log-inactivation was observed compared to the lower than 1 log-inactivation in the CT value of 4500 demonstrated for HP alone. These results clearly indicated the synergistic effect of Cu^++^ on HP performance. Finally, it can be said that, from the stand point of technical feasibility, a combination of HP and Cu^++^ could reduce the number of FC_s_ below the WHO guideline for irrigation in both RW and ASE. Further, in the case of ASE, it was possible to reduce FC_s_ to as low as the Iranian standard, i.e. 400 MPN/100 mL, which is required for irrigation. However, economical feasibility of the process should be also studied in future works.
